# Chromosomal instability in adult-type diffuse gliomas

**DOI:** 10.1186/s40478-022-01420-w

**Published:** 2022-08-17

**Authors:** Timothy E. Richardson, Jamie M. Walker, Kalil G. Abdullah, Samuel K. McBrayer, Mariano S. Viapiano, Zarmeen M. Mussa, Nadejda M. Tsankova, Matija Snuderl, Kimmo J. Hatanpaa

**Affiliations:** 1 Department of Pathology, Molecular, and Cell-Based Medicine, Icahn School of Medicine at Mount Sinai, Annenberg Building, 15th Floor, 1468 Madison Avenue, New York, NY 10029 USA; 2 Department of Neuroscience, Icahn School of Medicine at Mount Sinai, New York, NY 10029 USA; 3 Department of Neurosurgery, University of Pittsburgh School of Medicine, 200 Lothrop St, Pittsburgh, PA 15213 USA; 4 Hillman Comprehensive Cancer Center, University of Pittsburgh Medical Center, 5115 Centre Ave, Pittsburgh, PA 15232 USA; 5 Simmons Comprehensive Cancer Center, University of Texas Southwestern Medical Center, Dallas, TX 75390 USA; 6 Children’s Medical Center Research Institute, University of Texas Southwestern Medical Center, Dallas, TX 75390 USA; 7 Department of Neuroscience and Physiology, State University of New York, Upstate Medical University, Syracuse, NY 13210 USA; 8 Department of Neurosurgery, State University of New York, Upstate Medical University, Syracuse, NY 13210 USA; 9 Department of Pathology, New York University Langone Health, New York City, NY 10016 USA; 10 Department of Pathology, University of Texas Southwestern Medical Center, Dallas, TX 75390 USA

**Keywords:** Glioma, Astrocytoma, Oligodendroglioma, Glioblastoma, Chromothripsis, Chromosomal instability, CIN, Copy number burden, Copy number variation

## Abstract

Chromosomal instability (CIN) is a fundamental property of cancer and a key underlying mechanism of tumorigenesis and malignant progression, and has been documented in a wide variety of cancers, including colorectal carcinoma with mutations in genes such as *APC*. Recent reports have demonstrated that CIN, driven in part by mutations in genes maintaining overall genomic stability, is found in subsets of adult-type diffusely infiltrating gliomas of all histologic and molecular grades, with resulting elevated overall copy number burden, chromothripsis, and poor clinical outcome. Still, relatively few studies have examined the effect of this process, due in part to the difficulty of routinely measuring CIN clinically. Herein, we review the underlying mechanisms of CIN, the relationship between chromosomal instability and malignancy, the prognostic significance and treatment potential in various cancers, systemic disease, and more specifically, in diffusely infiltrating glioma subtypes. While still in the early stages of discovery compared to other solid tumor types in which CIN is a known driver of malignancy, the presence of CIN as an early factor in gliomas may in part explain the ability of these tumors to develop resistance to standard therapy, while also providing a potential molecular target for future therapies.

## Introduction

Diffuse glioma as a distinct entity was first identified microscopically and named in 1865 by Rudolf Virchow who designated two categories, roughly corresponding to “low grade” and “high grade”. Harvey Cushing and Percival Bailey first described “glioblastoma” in 1926, an entity that was subsequently refined by the observations of Hans-Joachim Scherer who distinguished between “primary” and “secondary” glioblastoma [32, 110]. Further refinement in diagnostic criteria came with electron microscope studies, followed by immunohistochemical (IHC) markers, and more recently molecular characterization of both low-grade and high-grade gliomas, although official neuropathologic diagnosis and grading were based primarily on histopathologic characteristics until 2016 [63, 64].

Currently, diffuse gliomas occur in approximately 16,600 individuals in the United States annually, representing 19.3% of all central nervous system (CNS) tumors at a rate of 4.52/100,000 individuals annually. The most malignant of these tumors, glioblastoma (WHO grade 4), is the most common form of diffuse glioma with a yearly incidence of approximately 12,000 cases in the United States (3.23/100,000 individuals), representing 14.3% of all intracranial tumors and 49.1% of all primary malignant CNS neoplasms. Despite advances in our understanding of the underlying pathogenesis of glioma and advances in treatment modalities, diffuse gliomas remain a surgically incurable disease, and the 5-year survival rate for glioblastoma remains approximately 6.8% (although this figure varies considerably by age group) [82], and many studies consider survival of more than 36 months to be “long-term survival” (LTS) in these patients [56, 93].

**Fig. 1:**
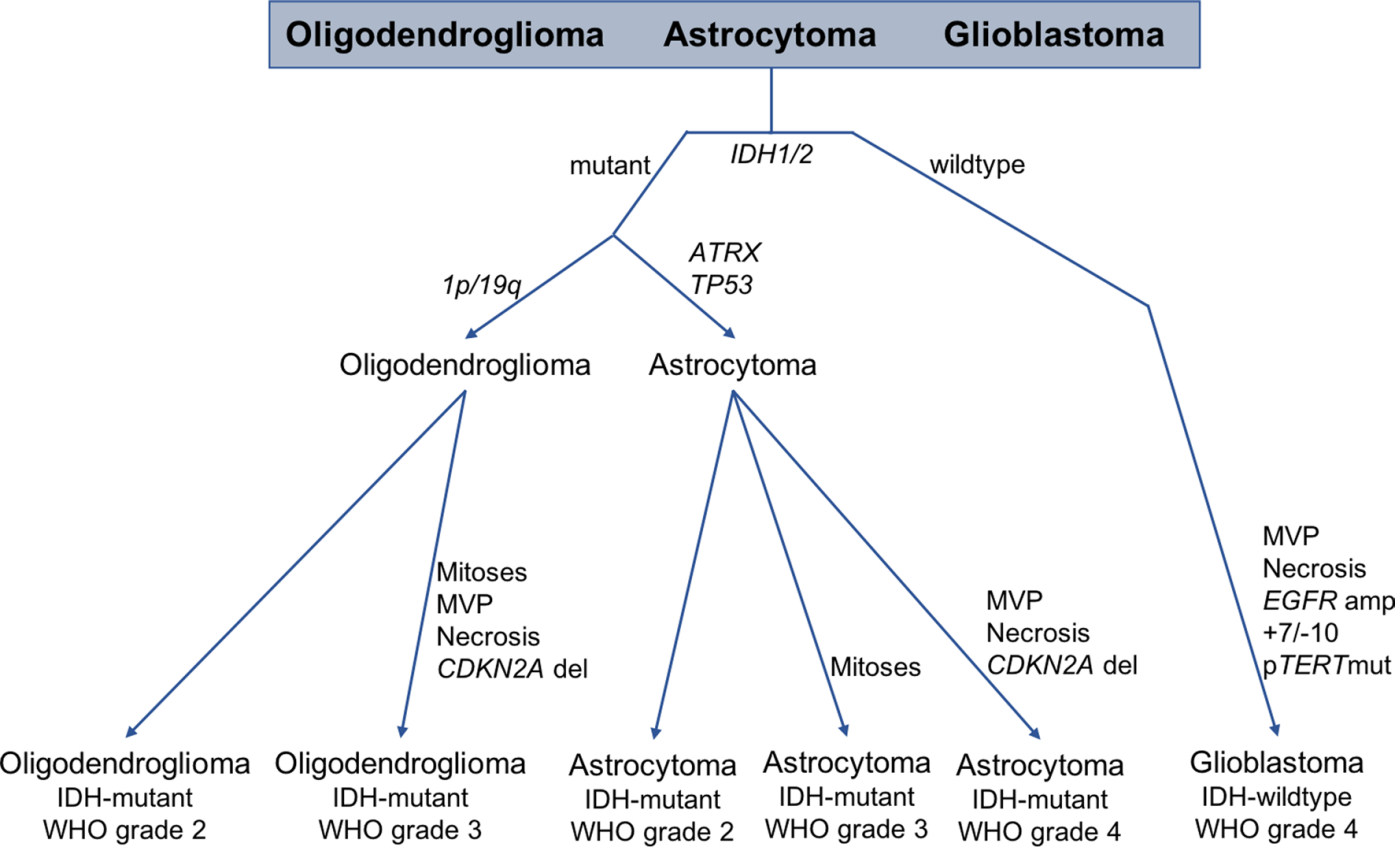
Diagnostic algorithm for integrating histologic and molecular features into a combined diffuse glioma diagnosis. Adapted from Louis et al. 2016 [64] and Louis et al. 2021 [65]

Beginning with the 2016 revised 4th Edition of the WHO Classification of Tumours of the Central Nervous System [64], diffusely infiltrating gliomas in adults have been subdivided and graded according to both histologic and molecular features, based on the findings of a number of large-scale, landmark studies [17, 19, 20, 23, 29, 34, 122]. This diagnostic system underwent further revision in 2021 [65] to more fully integrate molecular features into the neuropathologic definitions and terminology of these tumors (Fig. 1). Diffusely infiltrating IDH-wildtype gliomas tend to have the most aggressive behavior and worst clinical outcomes, and are designated as ‘Glioblastoma, IDH-wildtype’ if they have at least one of the following features: microvascular proliferation, necrosis, *EGFR* amplification, *TERT* promoter mutation, and/or simultaneous gain of chromosome 7 and loss of chromosome 10 (+ 7/− 10) [16, 65]. There remains evidence that a distinct category of lower-grade IDH-wildtype diffuse gliomas exists in adults with a more indolent clinical course, a “true” IDH-wildtype low-grade glioma [92], an assertion supported by recent methylome analysis [103]. There is also evidence that the absence of *EGFR* amplification, *TERT* promoter mutation, and + 7/− 10 is associated with a better clinical course in tumors that qualify as IDH-wildtype glioblastoma by histologic features alone [38]. In addition, there is debate as to the true impact of isolated *TERT* promoter mutations (those occurring without the traditional histologic features of glioblastoma, *EGFR* amplification, or + 7/− 10), particularly in tumors with grade 2 histology, suggesting that part of the effect of *TERT* promoter mutation may be due to their frequent co-occurrence with *EGFR* amplification, + 7/− 10, and/or more aggressive histologic features [8, 37, 44, 95].

Mutations in *IDH1* and *IDH2* (most commonly the *IDH1* R132H variant) define the majority of histologically lower-grade diffuse gliomas as well as what was previously termed “secondary glioblastoma” (i.e., tumors with grade 4 histology and documented radiologic and/or histopathologic evolution from lower-grade gliomas). Tumors with mutations in *IDH1* or *IDH2* and simultaneous loss of the entire chromosome arms 1p and 19q (and wildtype *ATRX* and *TP53*) are classified as ‘Oligodendroglioma, IDH-mutant and 1p/19q-codeleted’, and frequently have alterations in *CIC*, *FUBP1*, and the promoter region of *TERT* [50, 91, 101, 124]. These tumors are designated as WHO grade 3 if they have significant mitotic activity, microvascular proliferation, necrosis, or homozygous *CDKN2A* deletion, and are classified as WHO grade 2 in the absence of these findings.

Because the term “glioblastoma’ is now reserved for adult-type, WHO grade 4 diffuse gliomas lacking *IDH1/2* mutations, IDH-mutant tumors with retained 1p/19q (frequently with *ATRX* and/or *TP53* mutation) are designated as ‘Astrocytoma, IDH-mutant’ [15, 65]. Tumors in this category with “significant” mitotic activity are assigned to WHO grade 3, although an exact threshold of mitotic figures for clinical risk stratification has not been established [15, 81, 119, 125]. IDH-mutant astrocytomas are WHO grade 4 in the presence of microvascular proliferation, necrosis, and/or homozygous *CDKN2A* deletion [15, 65, 90], although other molecular features have been suggested as well [3, 15, 29, 70, 105]. IDH-mutant astrocytomas without these histologic or molecular features are designated as WHO grade 2. Given the lack of strong evidence that WHO grade 3 is associated with a significantly worse patient outcome compared with WHO grade 2, IDH-mutant astrocytomas grades 2–3 are now often pooled into the single category “lower-grade IDH-mutant astrocytoma” in both clinical practice and research.

Chromosomal instability (CIN) has been established as an underlying driver of malignancy in many different cancers [60, 118]. Due to recent evidence that it may be involved in the pathogenesis of some subsets of diffusely infiltrating gliomas it was considered by the Consortium to Inform Molecular and Practical Approaches to CNS Tumor Taxonomy (cIMPACT-NOW) panel in 2020 for possible inclusion in the diagnostic and grading criteria of IDH-mutant astrocytoma, however due to challenges in comparing between studies and lack of consensus on copy number variation (CNV) threshold, this feature was not endorsed at the time [15]. In this review, we discuss the definition, underlying mechanisms, and measurement methods of CIN, the concept of CIN as a molecular process driving tumorigenesis and malignant progression of solid tumors and other diseases, and the presence and consequence of this feature in a subset of diffuse gliomas within the context of recent changes to the WHO classification and diagnostic systems.

## Defining and measuring chromosomal instability

The majority of human cancers exhibit some form of genomic instability, which may take several forms but ultimately results in the ongoing and progressive accumulation of genetic defects, intercellular genomic heterogeneity, and tumor evolution. One such process, microsatellite instability (MSI), best characterized in colorectal cancer where it accounts for approximately 15% of cases, is a hypermutation phenotype resulting from inactivating mutation, deletion, or hypermethylation of DNA mismatch repair genes (*MLH1*, *MSH2*, *MSH6*, *PMS2*), which in turn results in rapid and unopposed accumulation of errors in DNA during replication [13, 61]. Mismatch repair deficiency leading to a high number of mutations in microsatellites (MSI-H) has also been identified in numerous other tumor types, including a recently described subtype of IDH-mutant astrocytoma [111], and has subsequently been found in additional glioma subgroups, in the context of constitutional mismatch repair deficiency syndrome, Li–Fraumeni syndrome, Cowden syndrome, Lynch syndrome, or sporadically [53, 54].

Chromosomal instability (CIN) is the other common form of genomic instability. The presence of numerical or structural alterations to chromosomes as a feature of cancer has been known for more than 100 years [14, 120], and CIN is a dynamic and progressive process that describes an ongoing, high rate of chromosomal abnormalities, largely through chromosomal mis-segregation, resulting in mounting cell-to-cell variability in chromosomal content [42, 118]. This process is frequently due to a mutation in one of a wide array of genes associated with structural chromosomal maintenance and mitotic control (Table 1) and tends to cause large-scale chromosomal damage [4, 24, 45], resulting in both numeric chromosomal changes and large-scale structural changes within chromosomes [46, 68]. This process can lead to the gain or loss of fragments or whole chromosomes within a single mitotic cycle, although it can also involve segmental aneuploidy, mutations, and copy number changes, as well as epigenetic structural changes [42]. In numerical CIN, there is more rapid gain and loss of whole chromosomes, resulting in variable aneuploidy, while in structural CIN, there is an increased rate of intra-chromosomal aberrations due to double stranded DNA breaks with potential rearrangement, resulting in gains or losses of chromosome segments, chromosomal fusion, mitotic recombination, and chromothripsis, producing a series of sub-clones with varying growth rates, malignant potential, resistance to therapy, tendency to invade and metastasize, among other phenotypes [4, 41, 45, 46, 78, 113]. Selective pressure is then applied to the resulting heterogeneous population of tumor cells, and more malignant and aggressive clones with a fitness advantage in the tumor microenvironment frequently become dominant by Darwinian mechanisms [18, 40, 80]. This mechanism may in part explain the relatively poor prognosis of that typically accompanies subsets of neoplasms with CIN [28, 48].

**Table 1:** Select genes associated with maintenance of chromosomal stability

*APC*	*FANCG*	*NBN*
*ATM*	*FANCI*	*NBS1*
*ATR*	*FANCJ (BRIP1)*	*PINX1*
*AURKA*	*FANCL*	*PLK1*
*AURKB*	*FANCM*	*POLB*
*BARD1*	*FANCN (PALB2)*	*POLK*
*BLM*	*FANCO (RAD51C)*	*POLN*
*BRCA1 (FANCS)*	*FANCP (SLX4)*	*RAD51 (FANCR)*
*BRCA2 (FANCD1)*	*FANCQ (ERCC4)*	*RAD52*
*BUB1B*	*FANCR (RAD51)*	*REV3*
*CCNE1*	*FANCS (BRCA1)*	*SMC1*
*CDC4 (FBXW7)*	*FANCT (UBE2T)*	*SNM1B*
*CHK1*	*FLJ10036*	*TERC*
*CLSPN*	*H2AFX*	*TERF1 (PIN2)*
*DNA-PK (PRKDC)*	*HUS1*	*TOP1*
*EME1*	*KIF11*	*TP53*
*FANCA*	*KIFC1*	*WRN*
*FANCB*	*KNTC1*	*XLF*
*FANCC*	*LIG4*	*ZW10*
*FANCD1 (BRCA2)*	*MAD2L1*	
*FANCD2*	*MPS1*	
*FANCE*	*MRE11A*	
*FANCF*	*MUS81*	

It is critical to note, however, that aneuploidy and structural chromosomal alterations may represent a measure of CIN, but are not synonymous with the process of CIN [88, 118]. Aneuploidy and structural alterations can result from CIN, however, aneuploidy can be static or stable in a number of disorders, including acute lymphoblastic leukemia [86], neuroblastoma [52], and oligodendroglioma [47, 89], as well as congenital conditions with underlying aneuploidy such as trisomy 21 [85]. In contrast, CIN as a process represents the rate of chromosomal change between cells over successive generations.

Chromosomal instability has perhaps best been described in colorectal carcinoma, in which it is present in approximately 85% of cases, which are characterized by mutations in *adenomatous polyposis coli* (*APC*) or *β-catenin* (*CTNNB1*) genes in both sporadic and hereditary forms [74, 75, 108]. CIN appears to be an early event in polyp formation that is followed by malignant transformation with additional alterations in oncogenes and tumor suppressor genes, some of which may result from CIN-related mechanisms [36, 66, 121]. Since the discovery of *APC*, more than 100 genes have been identified to play a role in the maintenance of chromosomal stability, with functions centered around DNA repair, cell-cycle regulation, spindle assembly, mitotic fidelity, centrosome function and fidelity, cytokinesis, and mitotic checkpoints, among others, but due to the complexity of the cellular replication process, it has been hypothesized that mutations in up to 2,300 genes related to these processes may result in chromosomal instability [6, 109, 114, 118]. Additionally, many other tumor types have been shown to have CIN as an initiating event or as a significant contributor to tumor progression and malignancy, including lung and oral squamous cell carcinomas [102, 126], lung adenocarcinoma [28], breast carcinoma [112], endometrial carcinoma [76], and diffuse large B-cell lymphoma (DLBCL) [5], among numerous other cancers and non-neoplastic conditions, including Fanconi anemia, which may predispose patients to numerous types of malignancies [26]. Although there is a large set of genes in which mutations have been demonstrated to underlie CIN initiation in these various diseases, the frequency of CIN among such diverse cancers and the susceptibility of germline carriers to developing cancer suggests a common mechanism of tumor initiation and malignancy.

Because CIN is an ongoing process, detection can be difficult, particularly with CNS neoplasms, in which only a small biopsy may be available for genomic analysis, and so a number of direct and indirect measurement methods for detecting CIN have been proposed [42, 118]. The most direct method for identifying CIN involves the lengthy and labor-intensive process of determining the rate of new karyotype abnormalities in successive generations of cultured tumor cells [60, 61]. Additional direct methods for detecting chromosomal instability include assessment of cell-to-cell aneuploidy and chromosomal alterations with fluorescent in situ hybridization (FISH) analysis [28, 102, 112, 126] and newer technologies such as single cell comparative genomic hybridization (CGH) [42, 113] and single cell sequencing [69, 77, 84, 128] to determine genomic variation between cells in the same tumor at a single point in time. In patients with recurrent tumors or metastases, repeated assessment of whole genome sequencing and copy number profiling can provide information on temporal genomic evolution within the same tumor [104, 127]. Indirect methods include histologic features such as nuclear size and micronucleus formation [9, 10, 123], the presence of double minutes or circular extrachromosomal DNA (ecDNA) [1, 33, 100, 106], observation of anaphase segregation errors in fixed tissue [5], as well as evaluation of sets of genes with known functions correlated to chromosomal function during mitosis and mitotic checkpoints, genomic integrity and DNA damage, and overall DNA structural maintenance, or genes with otherwise altered expression levels in tumors with known CIN [22].

Though difficult to demonstrate in a single biopsy, identification of CIN in tumors in which it is present is crucial. While tumors with CIN generally tend to be more aggressive, more drug resistant, and have a worse clinical course than their chromosomally stable counterparts [42, 113, 118], CIN can also serve as a target for therapy in addition to identifying more aggressive cases which may benefit from more intensive therapy initially. There are already many categories of drugs with prior FDA approval or in clinical trials for other cancers that strategically either reduce or increase CIN in tumor cells [4, 113, 114]. These include kinetochore modifiers, microtubule stabilizers and destabilizers, mitotic checkpoint modifiers, chromatin modifiers, and centrosome modifiers to prevent multipolar spindle formation, among others [4, 113]. In general, CIN-reducing therapies inhibit or decrease cell division in the presence of DNA damage or chromosomal or mitotic abnormalities and/or lower the rate of chromosomal mis-segregation to prevent further damage, while CIN-inducing therapies take advantage of the natural inclination of the tumor cell to progressively accumulate chromosomal damage and push it past a threshold of cell viability, ultimately leading to cell death. The viability of this latter strategy is supported by the finding that tumors with the highest levels of CIN and the most rapid development of chromosomal alterations often respond better to therapy [11]. Other authors have urged caution with this approach as therapies which promote CIN may fail to induce death of all tumor cells and the artificially induced increase in CIN rate may promote a more malignant tumor with more metastatic potential or drug resistant properties [113].

## IDH-mutant astrocytoma

**Fig. 2:**
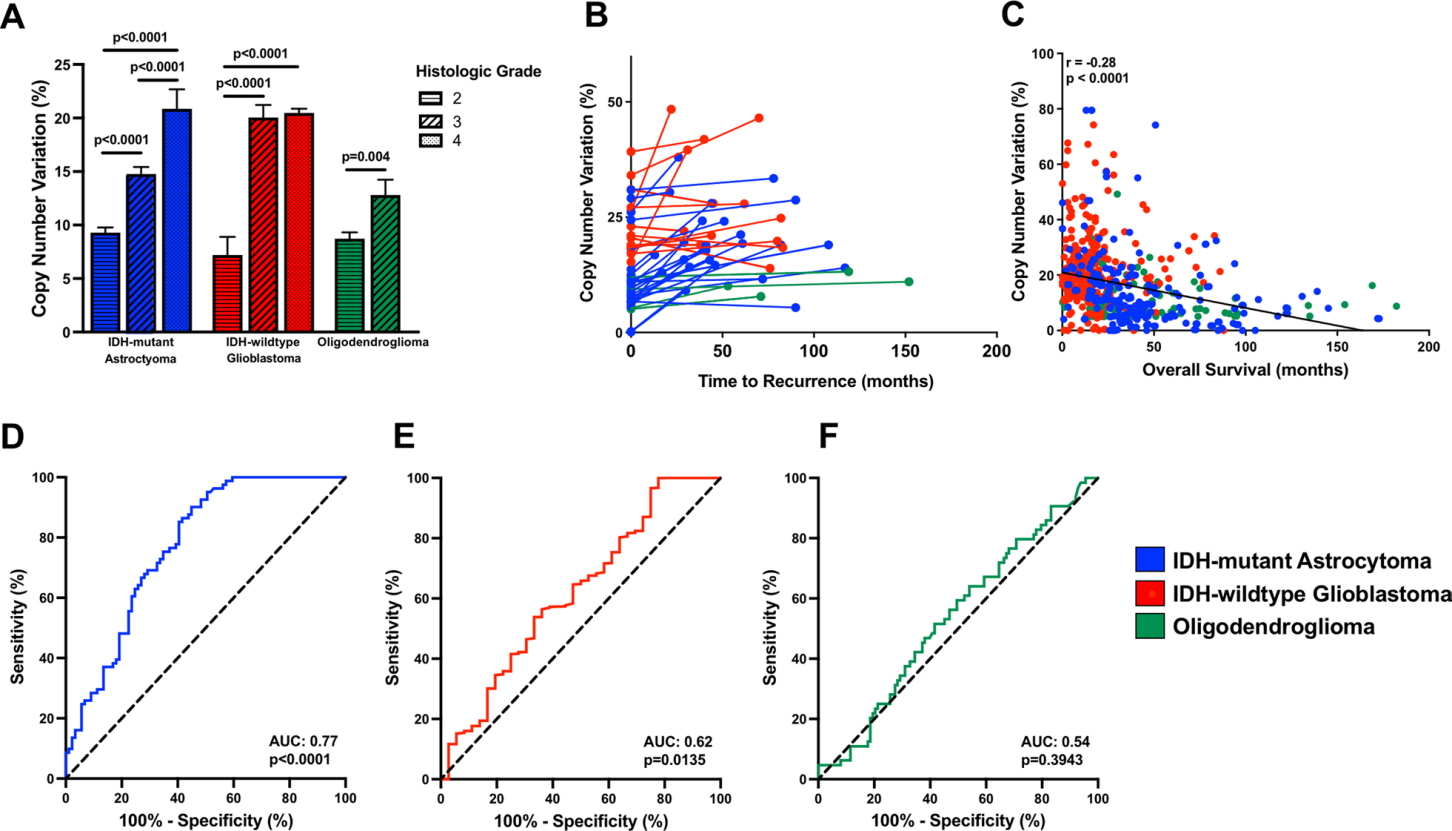
**A** Copy number variation (CNV) levels (expressed here as a percentage of the total genome) demonstrating a significant difference between overall CNV in all grades of IDH-mutant astrocytoma (*p* < 0.0001), between IDH-wildtype tumors histologically consistent with grade 2 and their histologic grade 3 and 4 counterparts (*p* < 0.0001), and between grade 2 and 3 oligodendroglioma (*p* = 0.0036), **B** copy number burden plot demonstrating a significant increase in the mean CNV in IDH-mutant astrocytoma (mean increase of 7.9 ± 1.1% in initial biopsy/resection versus recurrence; n = 22; *p* = 0.0012), and trend toward increased mean CNV percentage in IDH-wildtype glioblastoma (mean increase of 6.4 ± 1.1% in initial biopsy/resection versus recurrence; n = 13; *p* = 0.0795), **C** copy number burden plot demonstrating an inverse relationship between CNV in initial diffuse glioma biopsy and overall patient survival (r = -0.2507, *p* < 0.0001), and receiver operating characteristic (ROC) curves demonstrating the relative value of CNV in predicting outcome in **D** IDH-mutant astrocytoma (Area Under Curve (AUC) = 0.77; *p* < 0.0001), **E** IDH-wildtype glioblastoma (AUC = 0.62; *p* = 0.0135), and **F** oligodendroglioma (AUC = 0.54; *p* = 0.3943). All data are derived from Richardson et al. 2021 [97] and Liu et al. 2022 [62]

The presence and effect of chromosomal instability in adult diffuse gliomas is not as well understood as in other neoplasms, with relatively few studies examining the effects of CIN, chromothripsis, and mutations in genes with primary functions related to the maintenance of overall genomic stability, but the impact of overall copy number burden in IDH-mutant astrocytoma has been demonstrated in a number of different studies. Unlike IDH-wildtype glioblastoma or IDH-mutant and 1p/19q-codeleted oligodendroglioma, genomic identification of significant targets in cancer (GISTIC) algorithms highlight fewer chromosomal regions with well-defined and consistent alterations in IDH-mutant astrocytomas, instead showing a pattern of relatively random distribution of copy number alterations across the entire genome [71, 94, 96, 98]. The overall level of CNV increases with increasing grade in IDH-mutant astrocytoma (Fig. 2A) and oligodendroglioma as well as with malignant behavior in IDH-wildtype glioblastoma [30, 97, 99]. CNV has been shown to increase both over time and with increased physical distance of infiltrating cells in the same tumor, as subsequent resections and autopsy specimens tend to show increased levels of overall CNV compared to their initial biopsies (Fig. 2B), although it should also be noted that therapy between tumor sampling may alter the copy number profile [67].

**Fig. 3:**
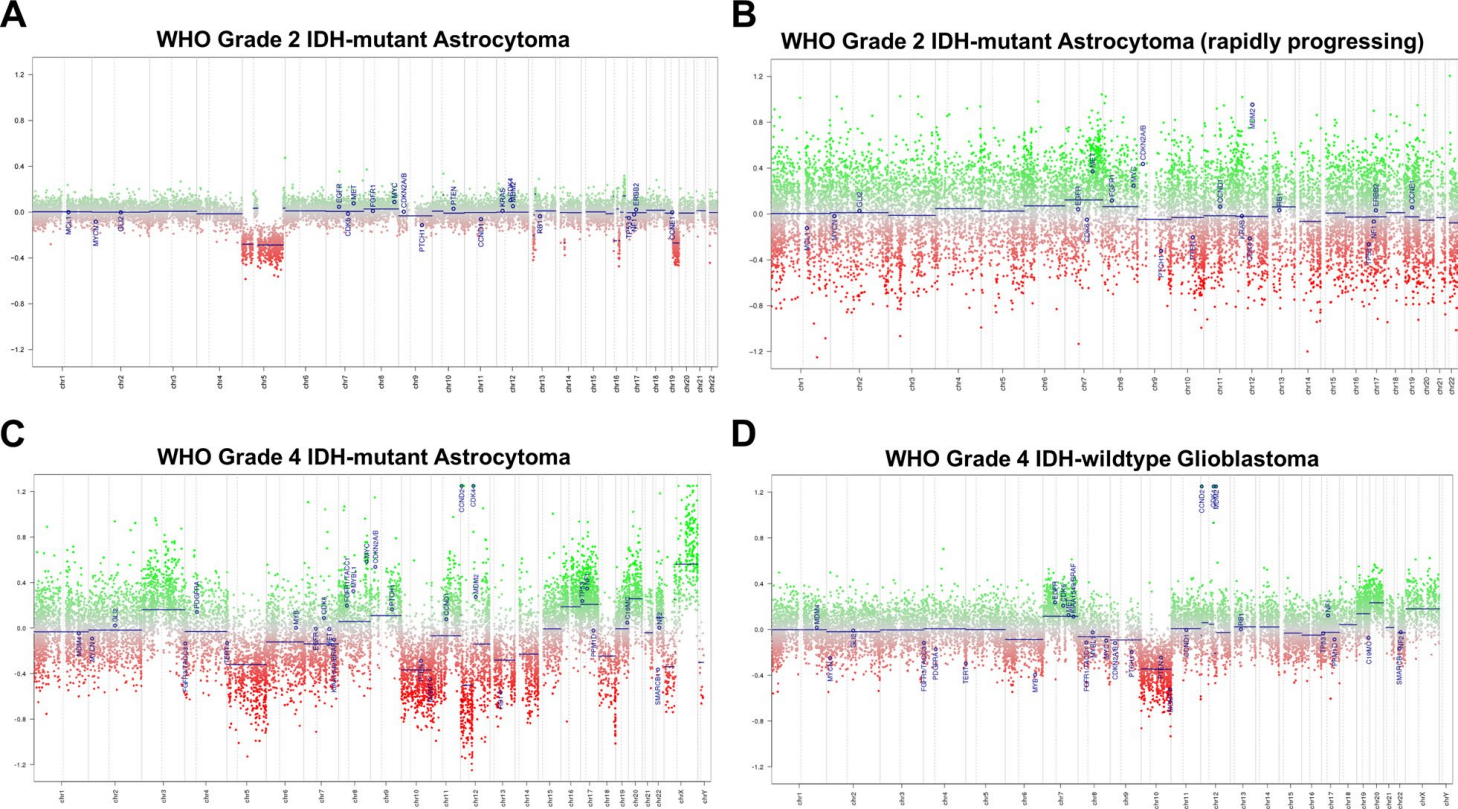
Representative copy number profiles of **A** a WHO grade 2 IDH-mutant astrocytoma case with late recurrence and overall patient survival of greater than 9 years (conventional clinical course), **B** a WHO grade 2 IDH-mutant astrocytoma with rapid progression to WHO grade 4 and short patient survival, **C** a WHO grade 4 IDH-mutant astrocytoma, and **D** a WHO grade 4 IDH-wildtype glioblastoma, for comparison. “Gain” or “loss” in the copy number profiles was defined as copy number change log2 ≥ 0.3. All cases represent the initial biopsy/resection specimen before any radiation or chemotherapy was given to the patient. Copy number plots for the IDH-mutant cases are reproduced from Richardson, et al. 2017 [98] (https://www.springer.com/journal/11060) and Richardson, et al. 2019 [94] (https://academic.oup.com/jnen) and are all used here with permission

CNV, distributed across the entire genome, is significantly elevated in lower-grade IDH-mutant astrocytomas with rapid progression and short overall patient survival intervals relative to grade-matched IDH-mutant astrocytomas with more conventional clinical courses, and in many cases their copy number plots are indistinguishable from or demonstrate even greater intra-chromosomal gains and losses than WHO grade 4 IDH-mutant astrocytoma (Fig. 3) [94, 96, 98]. This elevated copy number burden is found in IDH-mutant astrocytomas with poor clinical outcomes and with additional established poor prognostic molecular features, such as *CDKN2A* and *CDK4*, but is also found in cases where no other features suggestive of higher molecular grade are present [71, 96, 98]. These cases also have more frequent chromothripsis [30, 71, 78, 96]. Overall survival is inversely correlated with overall CNV level (Fig. 2C) and incongruously elevated CNV is found in the initial biopsies of lower-grade IDH-mutant astrocytomas selected exclusively for poor clinical outcomes and poor overall survival intervals [96, 98]. IDH-mutant astrocytomas have previously been successfully stratified exclusively by global CNV level at initial biopsy/resection with a threshold of 10–15% of the genome (approximately 310–470 Megabase pairs (Mbp) with copy number change log2 ≥ 0.3) [3, 72, 97, 105]. Receiver operating characteristic (ROC) curves utilizing 222 previously-analyzed lower-grade IDH-mutant astrocytomas demonstrate the best combined sensitivity and specificity at overall CNV levels between 12.5 and 15% (~ 387–470 Mbp) (Fig. 2D). These findings suggest that this chromosomal complexity/copy number burden pattern occurs during the progression to higher grade astrocytoma, may precede histologic progression, and may in part drive this progression, as well as serve as a useful molecular prognostic factor in otherwise histologically and molecularly low-grade astrocytoma cases.

**Fig. 4:**
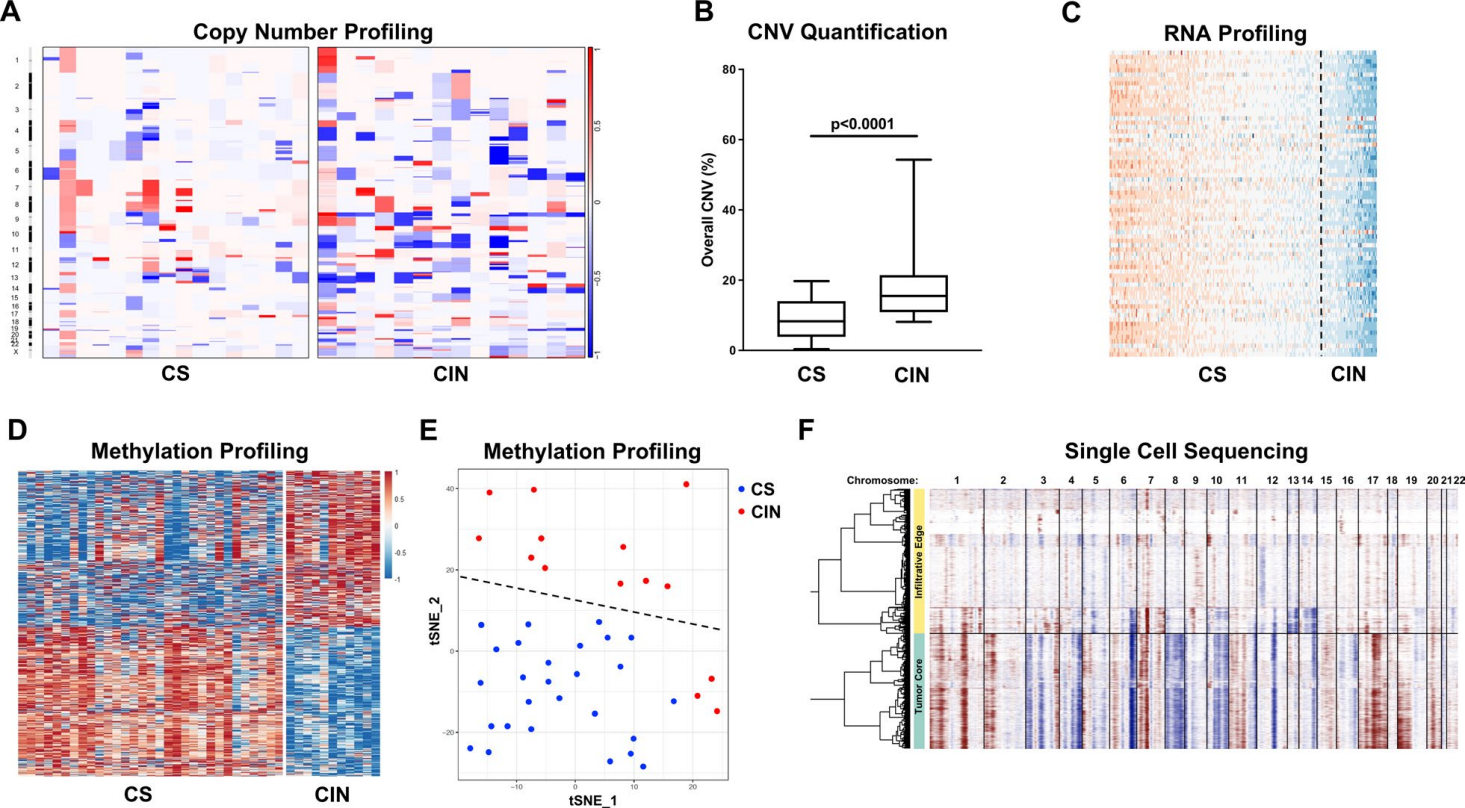
Examples of modern indirect and direct detection methods of chromosomal instability in cohorts of IDH-mutant astrocytoma. **A**, **B** Copy number profiles and copy number variation (CNV) quantification in otherwise low-grade tumors with chromosomal instability (CIN) versus relatively chromosomally stable tumors (CS), derived from Illumina Infinium DNA methylation 450 k and EPIC (850 k) arrays **C** mRNA expression profiling (CIN70 panel), **D**, **E** methylation profile-based clustering, derived from Illumina Infinium DNA methylation 450 k arrays, and **F** single nucleus RNA sequencing (InferCNV). Panels A and C are adapted from Richardson, et al. 2018 [96] and Richardson et al. 2021 [97], respectively (https://academic.oup.com/jnen), and Panels D-E are adapted from Liu et al. 2022 [62] (https://actaneurocomms.biomedcentral.com/), and are all used here with permission

Other molecular surrogates for CIN have been developed to indirectly identify the presence of CIN in solid tumors, including IDH-mutant astrocytoma cohorts (Fig. 4). In 2006, Carter et al. [22] identified 25- and 70-gene mRNA signatures (CIN25 and CIN70, respectively) that were consistently elevated in cases with previously demonstrated CIN, then applied that to numerous other solid tumor cohorts, including uncategorized glioma cases. In IDH-mutant astrocytomas, these gene panels were able to reproducibly identify a subset of IDH-mutant astrocytomas (27.4%) with evidence of CIN, which corresponded to significantly elevated copy number burden at initial biopsy/resection (irrespective of WHO grade), and significantly reduced progression-free survival (PFS) and overall survival (OS) intervals [97]. Using a similar strategy, we identified 14 IDH-mutant astrocytomas with prior evidence of CIN by at least two detection methods and 28 with no evidence of CIN, and performed methylation profiling to separate these cases into two distinct clusters based on the most differently methylated probes [62]. When this same methylome analysis was subsequently applied to a cohort of 245 IDH-mutant astrocytomas from The Cancer Genome Atlas (TCGA), two separate clusters were identified: one comprising 57 cases with significantly higher levels of CNV (21.2% vs. 7.4%), other evidence of CIN in the initial biopsy/resection, and worse PFS (median survival of 38 vs. 62 months) and OS (51 vs. 98 months) compared to a cluster of 188 cases that had lower CNV and better clinical outcomes. These data indicate that methylation profiling characteristics may be able to identify IDH-mutant astrocytoma with CNV based on a single biopsy specimen, in agreement with previous associations in other tumor types suggesting a link between DNA methylation status and chromosomal instability [35]. This feature is particularly promising, considering that DNA methylation profiling has been extensively validated for use as a diagnostic modality for other aspects of CNS neoplasms [21, 39, 83, 87].

Additionally, mutations in numerous genes with known functions related to maintaining chromosomal stability in many tumor types (Table 1) [114] have been identified in approximately 10% IDH-mutant astrocytomas, and mutations in these genes are significantly more frequent in cases with elevated CNV and poor clinical outcomes [71, 96, 97]. IDH-mutant astrocytoma cohorts can also be stratified into relatively good and poor survival outcomes based on this feature alone [97]. There remains a need for larger, comprehensive single cell sequencing studies (Fig. 4F) in varying grades of IDH-mutant astrocytoma to positively correlate more indirect markers of CIN and to determine high and low levels of CIN within this diffuse glioma subgroup, as well as consensus by expert molecular neuropathologists to set usefully thresholds for CNV level, methylation profiling characteristics, and mRNA expression levels that can be applied in the clinical setting.

## IDH-wildtype glioblastoma

The vast majority of diffuse IDH-wildtype tumors in adults have either histologic or molecular features of glioblastoma and resultant poor clinical outcomes [16, 65, 71]. These tumors have elevated overall CNV relative to IDH-mutant astrocytomas when expressed as a total percent of the genome [71, 97], however other studies have found that IDH-wildtype glioblastomas do not have significantly elevated CNV counts [30]. This may be due in part to differing methods of measurement and quantification thresholds, as well as the presence of “built-in”, discrete areas of CNV (including definitional + 7/− 10) that many IDH-wildtype glioblastomas have, regardless of their histologic features (Fig. 3D). There is, however, a small subset of IDH-wildtype diffuse gliomas that lack the histologic and molecular features of glioblastoma [16] as well as other high grade molecular features (including homozygous *CDKN2A* loss) that also had incongruously low overall CNV and relatively favorable clinical outcomes [92]. These IDH-wildtype tumors can be stratified by copy number burden (using 10% as a threshold), although the vast majority of both histologic and molecular glioblastomas do not fit into this category [97], and CNV level alone is not as useful of a measure in IDH-wildtype compared to IDH-mutant astrocytomas (Fig. 2E).

Other studies have shown that there are different histologic and genetic characteristics in primary glioblastoma compared to recurrences as well as between primary glioblastoma and rare metastases [2, 43, 55, 104], also suggesting accumulation of molecular alterations over time and in more spatially distant tumor foci. Single cell techniques have identified significant genomic and transcriptional diversity between IDH-wildtype glioblastoma cells of the same specimen. This diversity includes differential expression at the RNA level, different mutations, transcriptomic subtypes and epigenetic alterations constituting significant intratumoral heterogeneity, particularly within the population of glioma cancer stem cells, which correlates with particular gene signatures that are associated with differences in patient survival [31, 79, 84, 128]. These cell-to-cell changes may occur as the result of similar mechanisms as other cancers, including mutations in genes with roles in DNA repair and mitotic checkpoints, as well as interaction with the microenvironment and post-therapy changes. One notable example are rare gliomas with germline or somatic mutations in DNA polymerase E or D1 (*POLE* and *POLD1*) genes, enzymes normally involved in DNA replication and proofreading/repair, which result in chromosomal instability and extreme hypermutated phenotypes [27, 53]. These studies demonstrate genomic heterogeneity with resulting distinct sub-clonal populations within glioblastomas, with similar implications to other tumor types.

Histologic observation has long demonstrated increased variation in tumor nucleus size in more aggressive and higher grade CNS tumors, including glioblastoma, and more recent studies have demonstrated a high frequency of double minutes [33, 106] and micronucleus formation in some subsets of glioblastoma [7, 12], a feature associated with CIN in other solid cancers [9, 10, 123]. Recurrent glioblastomas also display significantly altered genomic profiles after treatment [51, 55], and differing expression of targetable cellular receptors and other molecular pathways, suggesting that this temporal heterogeneity may be affected by clinical treatment, which may in turn have implications for future therapy [104]. IDH-wildtype glioblastoma can also be stratified based on CIN70 mRNA panels, with high-CIN70 expressing tumors demonstrating significantly higher overall CNV at initial resection with worse PFS and OS. Notably, the majority of IDH-wildtype cases have high-CIN70 expression patterns (72.4%), unlike IDH-mutant astrocytomas, which coincides with their generally higher copy number burden at initial presentation, as expressed as a percentage of the total genome [97]. Mutations in genes with functions related to maintaining chromosomal stability occur in approximately 8% of IDH-wildtype glioblastomas as well, and while these cases have higher levels of CNV at initial resection, no significant difference in clinical outcome was identified [71, 97].

## Oligodendroglioma & other CNS neoplasms

Although some studies have identified polysomy, as defined as 2 or more signals for 1q and 19p, as a poor prognostic factor in oligodendroglioma cohorts [25, 107], there is less evidence that chromosomal instability plays a role in a significant number of oligodendroglioma cases. Like IDH-wildtype glioblastoma, oligodendroglioma has a built-in, definitional copy number alteration (whole-arm 1p/19q co-deletion, accounting for loss of approximately 5.1% of the genome) [47, 89]. Previous studies evaluating the role of CNV in oligodendroglioma have shown that CNV increases significantly from WHO grade 2 to 3 (Fig. 2A) [97, 99], however this does not appear to be an independent prognostic factor in this tumor type, and no useful CNV threshold by which to stratify oligodendroglioma has been established (Fig. 2F). In addition, no significant progression-free or overall survival differences were noted by stratifying oligodendroglioma by CIN70 mRNA profiling levels or by the presence or absence of mutations in genes with functions related to maintenance of chromosomal stability, although oligodendroglioma can be successfully stratified based on tumor mutation burden (TMB) [97, 99]. Furthermore, small single cell sequencing studies have shown that while there is clonal evolution and a population of undifferentiated cancer stem cells in some cases of oligodendroglioma, no evidence of chromosomal instability was identified [115, 117]. Chromosomal instability and chromothripsis have also been implicated in the initiation of other CNS tumors, including medulloblastomas, a subset of which occur as part of the Fanconi anemia spectrum [49, 58, 73, 116], and some other embryonal neoplasms [57]. Other types of classically aggressive CNS neoplasms appear not to involve significant CIN but are instead driven by distinct mutations leading to chromatin remodeling at the epigenetic level [59].

## Conclusions

Chromosomal instability and mutations in genes that are involved in guarding against large-scale genetic abnormalities are well known and well characterized in many systemic tumor types. In these tumors, research into the underlying cause of chromosomal instability, mechanisms of chromosomal alterations, and the contribution of chromosomal instability to tumorigenesis and tumor progression has yielded significant insight into cellular regulatory systems, mechanisms of cancer formation, and potential treatments targeting these changes. The impact of mutations in this set of genes and the resulting chromosomal damage are not yet well defined in gliomas. However, new insight from studying large groups of glioma patients has demonstrated that overall CNV changes and other genetic and epigenetic factors associated with chromosomal instability correlate with some previously known prognostic factors, including histologic grade and newer molecular features, and also have an effect on the clinical outcome within and across previously established glioma subgroups and grades, even in the absence of these other prognostic factors. This effect is most pronounced in IDH-mutant astrocytomas, in which it acts as an independent prognostic factor, and in some studies has significantly better prognostic utility than current WHO grading schemes, especially when correlating multiple measures of CIN. While detection of CIN remains challenging at the clinical level, recent advances in molecular diagnostic techniques provide opportunities to better understand this phenomenon. In particular, detecting CIN by CNV, DNA methylation, and/or gene expression profiles could provide a reliable guide for identifying gliomas driven by this molecular process, as in other solid tumor types. CIN deserves consideration as an underlying driver of tumor progression and tumor aggressiveness in gliomas, and could provide a therapeutic target for these surgically incurable tumors in the future.

## References

[1] Al-Rawi DH, Bakhoum SF (2022). Chromosomal instability as a source of genomic plasticity. Curr Opin Genet Dev.

[2] Anderson KJ, Tan AC, Parkinson J, Back M, Kastelan M, Newey A, Brewer J, Wheeler H, Hudson AL, Amin SB (2020). Molecular and clonal evolution in recurrent metastatic gliosarcoma. Cold Spring Harb Mol Case Stud.

[3] Aoki K, Nakamura H, Suzuki H, Matsuo K, Kataoka K, Shimamura T, Motomura K, Ohka F, Shiina S, Yamamoto T (2018). Prognostic relevance of genetic alterations in diffuse lower-grade gliomas. Neuro Oncol.

[4] Bakhoum SF, Compton DA (2012). Chromosomal instability and cancer: a complex relationship with therapeutic potential. J Clin Invest.

[5] Bakhoum SF, Danilova OV, Kaur P, Levy NB, Compton DA (2011). Chromosomal instability substantiates poor prognosis in patients with diffuse large B-cell lymphoma. Clin Cancer Res.

[6] Bakhoum SF, Thompson SL, Manning AL, Compton DA (2009). Genome stability is ensured by temporal control of kinetochore-microtubule dynamics. Nat Cell Biol.

[7] Ballester LY, Boghani Z, Baskin DS, Britz GW, Olsen R, Fuller GN, Powell SZ, Cykowski MD (2018). Creutzfeldt astrocytes may be seen in IDH-wildtype glioblastoma and retain expression of DNA repair and chromatin binding proteins. Brain Pathol.

[8] Berzero G, Di Stefano AL, Ronchi S, Bielle F, Villa C, Guillerm E, Capelle L, Mathon B, Laurenge A, Giry M (2021). IDH-wildtype lower-grade diffuse gliomas: the importance of histological grade and molecular assessment for prognostic stratification. Neuro Oncol.

[9] Bhatia A, Kumar Y (2013). Cancer cell micronucleus: an update on clinical and diagnostic applications. APMIS.

[10] Bhatia A, Kumar Y (2014). Relevance of microscopic indicators of chromosomal instability in routine reporting of malignancies. Diagn Cytopathol.

[11] Birkbak NJ, Eklund AC, Li Q, McClelland SE, Endesfelder D, Tan P, Tan IB, Richardson AL, Szallasi Z, Swanton C (2011). Paradoxical relationship between chromosomal instability and survival outcome in cancer. Cancer Res.

[12] Boghani Z, Steele WJ, Cykowski MD, Ballester LY, Britz G (2017). Creutzfeldt cell rich glioblastoma: a diagnostic dilemma. Cureus.

[13] Boland CR, Goel A (2010). Microsattelite instability in colorectal cancer. Gastroenterology.

[14] Boveri T (2008). Concerning the origin of malignant tumours by Theodor Boveri. Translated and annotated by Henry Harris. J Cell Sci.

[15] Brat DJ, Aldape K, Colman H, Figrarella-Branger D, Fuller GN, Giannini C, Holland EC, Jenkins RB, Kleinschmidt-DeMasters B, Komori T (2020). cIMPACT-NOW update 5: recommended grading criteria and terminologies for IDH-mutant astrocytomas. Acta Neuropathol.

[16] Brat DJ, Aldape K, Colman H, Holland EC, Louis DN, Jenkins RB, Kleinschmidt-DeMasters BK, Perry A, Reifenberger G, Stupp R (2018). cIMPACT-NOW update 3: recommended diagnostic criteria for "Diffuse astrocytic glioma, IDH-wildtype, with molecular features of glioblastoma, WHO grade IV". Acta Neuropathol.

[17] Brennan CW, Verhaak RG, McKenna A, Campos B, Noushmehr H, Salama SR, Zheng S, Chakravarty D, Sanborn JZ, Berman SH (2013). The somatic genomic landscape of glioblastoma. Cell.

[18] Cahill DP, Kinzler KW, Vogelstein B, Lengauer C (1999). Genetic instability and Darwinian selection in tumours. Trends Cell Biol.

[19] Cancer Genome Atlas Research N (2008). Comprehensive genomic characterization defines human glioblastoma genes and core pathways. Nature.

[20] Brat DJ, Verhaak RG, Aldape KD, Yung WK, Salama SR, Cooper LA, Rheinbay E, Miller CR, Vitucci M, Cancer Genome Atlas Research N (2015). Comprehensive, integrative genomic analysis of diffuse lower-grade gliomas. N Engl J Med.

[21] Capper D, Jones DTW, Sill M, Hovestadt V, Schrimpf D, Sturm D, Koelsche C, Sahm F, Chavez L, Reuss DE (2018). DNA methylation-based classification of central nervous system tumours. Nature.

[22] Carter SL, Eklund AC, Kohane IS, Harris LN, Szallasi Z (2006). A signature of chromosomal instability inferred from gene expression profiles predicts clinical outcome in multiple human cancers. Nat Genet.

[23] Ceccarelli M, Barthel FP, Malta TM, Sabedot TS, Salama SR, Murray BA, Morozova O, Newton Y, Radenbaugh A, Pagnotta SM (2016). Molecular profiling reveals biologically discrete subsets and pathways of progression in diffuse glioma. Cell.

[24] Chan SH, Ngeow J (2017). Germline mutation contribution to chromosomal instability. Endocr Relat Cancer.

[25] Chen H, Thomas C, Munoz FA, Alexandrescu S, Horbinski CM, Olar A, McGuone D, Camelo-Piragua S, Wang L, Pentsova E (2019). Polysomy is associated with poor outcome in 1p/19q codeleted oligodendroglial tumors. Neuro Oncol.

[26] Cheung RS, Taniguchi T (2017). Recent insights into the molecular basis of *Fanconi anemia*: genes, modifiers, and drivers. Int J Hematol.

[27] Cho B, Bryce C, Tsankova N (2021). A case of POLE mutation in high-grade astrocytoma with variant IDH1 mutation R132C and ultramutant phenotype. J Neuropathol Exp Neurol.

[28] Choi CM, Seo KW, Jang SJ, Oh YM, Shim TS, Kim WS, Lee DS, Lee SD (2009). Chromosomal instability is a risk factor for poor prognosis of adenocarcinoma of the lung: fluorescence in situ hybridization analysis of paraffin-embedded tissue from Korean patients. Lung Cancer.

[29] Cimino PJ, Zager M, McFerrin L, Wirsching HG, Bolouri H, Hentschel B, von Deimling A, Jones D, Reifenberger G, Weller M (2017). Multidimensional scaling of diffuse gliomas: application to the 2016 World Health Organization classification system with prognostically relevant molecular subtype discovery. Acta Neuropathol Commun.

[30] Cohen A, Sato M, Aldape K, Mason CC, Alfaro-Munoz K, Heathcock L, South ST, Abegglen LM, Schiffman JD, Colman H (2015). DNA copy number analysis of Grade II-III and Grade IV gliomas reveals differences in molecular ontogeny including chromothripsis associated with IDH mutation status. Acta Neuropathol Commun.

[31] Darmanis S, Sloan SA, Croote D, Mignardi M, Chernikova S, Samghababi P, Zhang Y, Neff N, Kowarsky M, Caneda C (2017). Single-cell RNA-seq analysis of infiltrating neoplastic cells at the migrating front of human glioblastoma. Cell Rep.

[32] DeAngelis LM, Mellinghoff IK (2011). Virchow 2011 or how to ID(H) human glioblastoma. J Clin Oncol.

[33] deCarvalho AC, Kim H, Poisson LM, Winn ME, Mueller C, Cherba D, Koeman J, Seth S, Protopopov A, Felicella M (2018). Discordant inheritance of chromosomal and extrachromosomal DNA elements contributes to dynamic disease evolution in glioblastoma. Nat Genet.

[34] Eckel-Passow JE, Lachance DH, Molinaro AM, Walsh KM, Decker PA, Sicotte H, Pekmezci M, Rice T, Kosel ML, Smirnov IV (2015). Glioma groups based on 1p/19q, IDH, and TERT promoter mutations in tumors. N Engl J Med.

[35] Eden A, Gaudet F, Waghmare A, Jaenisch R (2003). Chromosomal instability and tumors promoted by DNA hypomethylation. Science.

[36] Fearon ER, Vogelstein B (1990). A genetic model for colorectal tumorigenesis. Cell.

[37] Fujimoto K, Arita H, Satomi K, Yamasaki K, Matsushita Y, Nakamura T, Miyakita Y, Umehara T, Kobayashi K, Tamura K (2021). TERT promoter mutation status is necessary and sufficient to diagnose IDH-wildtype diffuse astrocytic glioma with molecular features of glioblastoma. Acta Neuropathol.

[38] Galbraith K, Kumar A, Abdullah KG, Walker JM, Adams SH, Prior T, Dimentberg R, Henderson FC, Mirchia K, Sathe AA (2020). Molecular correlates of long survival in IDH-wildtype glioblastoma cohorts. J Neuropathol Exp Neurol.

[39] Galbraith K, Snuderl M (2022). DNA methylation as a diagnostic tool. Acta Neuropathol Commun.

[40] Gao C, Furge K, Koeman J, Dykema K, Su Y, Cutler ML, Werts A, Haak P, Vande Woude GF (2007). Chromosome instability, chromosome transcriptome, and clonal evolution of tumor cell populations. Proc Natl Acad Sci USA.

[41] Gao C, Su Y, Koeman J, Haak E, Dykema K, Essenberg C, Hudson E, Petillo D, Khoo SK, Vande Woude GF (2016). Chromosome instability drives phenotypic switching to metastasis. Proc Natl Acad Sci USA.

[42] Geigl JB, Obenauf AC, Schwarzbraun T, Speicher MR (2008). Defining 'chromosomal instability'. Trends Genet.

[43] Georgescu MM, Olar A (2020). Genetic and histologic spatiotemporal evolution of recurrent, multifocal, multicentric and metastatic glioblastoma. Acta Neuropathol Commun.

[44] Giannini C, Giangaspero F (2021). TERT promoter mutation: is it enough to call a WHO grade II astrocytoma IDH wild-type glioblastoma?. Neuro Oncol.

[45] Gollin SM (2005). Mechanisms leading to chromosomal instability. Semin Cancer Biol.

[46] Gordon DJ, Resio B, Pellman D (2012). Causes and consequences of aneuploidy in cancer. Nat Rev Genet.

[47] Griffin CA, Burger P, Morsberger L, Yonescu R, Swierczynski S, Weingart JD, Murphy KM (2006). Identification of der(1;19)(q10;p10) in five oligodendrogliomas suggests mechanism of concurrent 1p and 19q loss. J Neuropathol Exp Neurol.

[48] Heilig CE, Löffler H, Mahlknecht U, Janssen JWG, Ho AD, Jauch A, Krämer A (2010). Chromosomal instability correlates with poor outcome in patients with myelodysplastic syndromes irrespectively of the cytogenetic risk group. J Cell Mol Med.

[49] Hirsch B, Shimamura A, Moreau L, Baldinger S, Hag-alshiekh M, Bostrom B, Sencer S, D'Andrea AD (2004). Association of biallelic BRCA2/FANCD1 mutations with spontaneous chromosomal instability and solid tumors of childhood. Blood.

[50] Jiao Y, Killela PJ, Reitman ZJ, Rasheed AB, Heaphy CM, de Wilde RF, Rodriguez FJ, Rosemberg S, Oba-Shinjo SM, Nagahashi Marie SK (2012). Frequent ATRX, CIC, FUBP1 and IDH1 mutations refine the classification of malignant gliomas. Oncotarget.

[51] Jonsson P, Lin AL, Young RJ, DiStefano NM, Hyman DM, Li BT, Berger MF, Zehir A, Ladanyi M, Solit DB (2019). Genomic Correlates of Disease Progression and Treatment Response in Prospectively Characterized Gliomas. Clin Cancer Res.

[52] Kaneko Y, Knudson AG (2000). Mechanism and relevance of ploidy in neuroblastoma. Genes Chromosom Cancer.

[53] Kim B, Tabori U, Hawkins C (2020). An update on the CNS manifestations of brain tumor polyposis syndromes. Acta Neuropathol.

[54] Kim H, Lim KY, Park JW, Kang J, Won JK, Lee K, Shim Y, Park CK, Kim SK, Choi SH (2022). Sporadic and Lynch syndrome-associated mismatch repair-deficient brain tumors. Lab Invest.

[55] Kim J, Lee IH, Cho HJ, Park CK, Jung YS, Kim Y, Nam SH, Kim BS, Johnson MD, Kong DS (2015). Spatiotemporal evolution of the primary glioblastoma genome. Cancer Cell.

[56] Krex D, Klink B, Hartmann C, von Deimling A, Pietsch T, Simon M, Sabel M, Steinbach JP, Heese O, Reifenberger G (2007). Long-term survival with glioblastoma multiforme. Brain.

[57] Lambo S, Grobner SN, Rausch T, Waszak SM, Schmidt C, Gorthi A, Romero JC, Mauermann M, Brabetz S, Krausert S (2019). The molecular landscape of ETMR at diagnosis and relapse. Nature.

[58] Lang PY, Nanjangud GJ, Sokolsky-Papkov M, Shaw C, Hwang D, Parker JS, Kabanov AV, Gershon TR (2016). ATR maintains chromosomal integrity during postnatal cerebellar neurogenesis and is required for medulloblastoma formation. Development.

[59] Lee RS, Roberts CW (2013). Rhabdoid tumors: an initial clue to the role of chromatin remodeling in cancer. Brain Pathol.

[60] Lengauer C, Kinzler KW, Vogelstein B (1998). Genetic instabilities in human cancers. Nature.

[61] Lengauer C, Kinzler KW, Vogelstein B (1997). Genetic instability in colorectal cancers. Nature.

[62] Liu Y, Sathe AA, Abdullah KG, McBrayer SK, Adams SH, Brenner AJ, Hatanpaa KJ, Viapiano MS, Xing C, Walker JM (2022). Global DNA methylation profiling reveals chromosomal instability in IDH-mutant astrocytomas. Acta Neuropathol Commun.

[63] Louis DN, Ohgaki H, Wiestler OD, Cavenee WK, Burger PC, Jouvet A, Scheithauer BW, Kleihues P (2007). The 2007 WHO classification of tumours of the central nervous system. Acta Neuropathol.

[64] Louis DN, Perry A, Reifenberger G, von Deimling A, Figarella-Branger D, Cavenee WK, Ohgaki H, Wiestler OD, Kleihues P, Ellison DW (2016). The 2016 World Health Organization classification of tumors of the central nervous system: a summary. Acta Neuropathol.

[65] Louis DN, Perry A, Wesseling P, Brat DJ, Cree IA, Figarella-Branger D, Hawkins C, Ng HK, Pfister SM, Reifenberger G (2021). The 2021 WHO Classification of tumors of the central nervous system: a summary. Neuro Oncol.

[66] Luebeck EG, Moolgavkar SH (2002). Multistage carcinogenesis and the incidence of colorectal cancer. Proc Natl Acad Sci USA.

[67] Lyon JF, Vasudevaraja V, Mirchia K, Walker JM, Corona RJ, Chin LS, Tran I, Snuderl M, Richardson TE, Viapiano MS (2021). Spatial progression and molecular heterogeneity of IDH-mutant glioblastoma determined by DNA methylation-based mapping. Acta Neuropathol Commun.

[68] McGranahan N, Burrell RA, Endesfelder D, Novelli MR, Swanton C (2012). Cancer chromosomal instability: therapeutic and diagnostic challenges. EMBO Rep.

[69] Meyer M, Reimand J, Lan X, Head R, Zhu X, Kushida M, Bayani J, Pressey JC, Lionel AC, Clarke ID (2015). Single cell-derived clonal analysis of human glioblastoma links functional and genomic heterogeneity. Proc Natl Acad Sci U S A.

[70] Mirchia K, Richardson TE (2020). Beyond IDH-mutation: emerging molecular diagnostic and prognostic features in adult diffuse gliomas. Cancers (Basel).

[71] Mirchia K, Sathe AA, Walker JM, Fudym Y, Galbraith K, Viapiano MS, Corona RJ, Snuderl M, Xing C, Hatanpaa KJ (2019). Total copy number variation as a prognostic factor in adult astrocytoma subtypes. Acta Neuropathol Commun.

[72] Mirchia K, Snuderl M, Galbraith K, Hatanpaa KJ, Walker JM, Richardson TE (2019). Establishing a prognostic threshold for total copy number variation within adult IDH-mutant grade II/III astrocytomas. Acta Neuropathol Commun.

[73] Mitani Y, Fukuoka K, Mori M, Arakawa Y, Matsushita Y, Hibiya Y, Honda S, Kobayashi M, Tanami Y, Kanemura Y (2021). Clinical aggressiveness of TP53-wild type Sonic Hedgehog medulloblastoma with MYCN amplification, chromosome 17p loss, and chromothripsis. J Neuropathol Exp Neurol.

[74] Morin PJ, Sparks AB, Korinek V, Barker N, Clevers H, Vogelstein B, Kinzler KW (1997). Activation of beta-catenin-Tcf signaling in colon cancer by mutations in beta-catenin or APC. Science.

[75] Muller MF, Ibrahim AE, Arends MJ (2016). Molecular pathological classification of colorectal cancer. Virchows Arch.

[76] Murayama-Hosokawa S, Oda K, Nakagawa S, Ishikawa S, Yamamoto S, Shoji K, Ikeda Y, Uehara Y, Fukayama M, McCormick F (2010). Genome-wide single-nucleotide polymorphism arrays in endometrial carcinomas associate extensive chromosomal instability with poor prognosis and unveil frequent chromosomal imbalances involved in the PI3-kinase pathway. Oncogene.

[77] Navin N, Kendall J, Troge J, Andrews P, Rodgers L, McIndoo J, Cook K, Stepansky A, Levy D, Esposito D (2011). Tumour evolution inferred by single-cell sequencing. Nature.

[78] Nazaryan-Petersen L, Bjerregaard VA, Nielsen FC, Tommerup N, Tumer Z (2020). Chromothripsis and DNA repair disorders. J Clin Med.

[79] Neftel C, Laffy J, Filbin MG, Hara T, Shore ME, Rahme GJ, Richman AR, Silverbush D, Shaw ML, Hebert CM (2019). An integrative model of cellular states, plasticity, and genetics for glioblastoma. Cell.

[80] Nowell PC (1976). The clonal evolution of tumor cell populations. Science.

[81] Olar A, Wani KM, Heathcock LE, van Thuijl HF, Gilbert MR, Armstrong TS, Sulman EP, Cahill DP, Vera-BOlanos E, Yuan Y (2015). IDH mutation status and role of WHO grade and mitotic index in overall survival in grade II–III diffuse gliomas. Acta Neuropathol.

[82] Ostrom QT, Cioffi G, Waite C, Kruchko C, Barnholtz-Sloan JS (2021). CBTRUS statistical report: primary brain and other central nervous system tumors diagnosed in the United States in 2014–2018. Neuro Oncol.

[83] Papanicolau-Sengos A, Aldape K (2022). DNA methylation profiling: an emerging paradigm for cancer diagnosis. Annu Rev Pathol.

[84] Patel AP, Tirosh I, Trombetta JJ, Shalek AK, Gillespie SM, Wakimoto H, Cahill DP, Nahed BV, Curry WT, Martuza RL (2014). Single-cell RNA-seq highlights intratumoral heterogeneity in primary glioblastoma. Science.

[85] Patterson D (2009). Molecular genetic analysis of Down syndrome. Hum Genet.

[86] Paulsson K, Johansson B (2009). High hyperdiploid childhood acute lymphoblastic leukemia. Genes Chromosomes Cancer.

[87] Pratt D, Sahm F, Aldape K (2021). DNA methylation profiling as a model for discovery and precision diagnostics in neuro-oncology. Neuro Oncol.

[88] Rajagopalan H, Nowak MA, Vogelstein B, Lengauer C (2003). The significance of unstable chromosomes in colorectal cancer. Nat Rev Cancer.

[89] Reifenberger J, Reifenberger G, Liu L, James CD, Wechsler W, Collins VP (1994). Molecular genetic analysis of oligodendroglial tumors shows preferential allelic deletions on 19q and 1p. Am J Pathol.

[90] Reis GF, Pekmezci M, Hansen HM, Rice T, Marshall RE, Molinaro AM, Phillips JJ, Vogel H, Wiencke JK, Wrensch MR (2015). CDKN2A loss is associated with shortened overall survival in lower-grade (World Health Organization Grades II-III) astrocytomas. J Neuropathol Exp Neurol.

[91] Reuss DE, Sahm F, Schrimpf D, Wiestler B, Capper D, Koelsche C, Schweizer L, Korshunov A, Jones DT, Hovestadt V (2015). ATRX and IDH1-R132H immunohistochemistry with subsequent copy number analysis and IDH sequencing as a basis for an "integrated" diagnostic approach for adult astrocytoma, oligodendroglioma and glioblastoma. Acta Neuropathol.

[92] Richardson TE, Hatanpaa KJ, Walker JM (2021). Molecular characterization of "true" low-grade IDH-wildtype astrocytomas. J Neuropathol Exp Neurol.

[93] Richardson TE, Kumar A, Xing C, Hatanpaa K, Walker JW (2020). Overcoming the odds: toward a molecular profile of long-term survival in glioblastoma. J Neuropathol Exp Neurol.

[94] Richardson TE, Patel S, Serrano J, Sathe AA, Daoud EV, Oliver D, Maher EA, Madrigales A, Mickey BE, Taxter T (2019). Genome-wide analysis of glioblastoma patients with unexpectedly long survival. J Neuropathol Exp Neurol.

[95] Richardson TE, Raghunathan A, Abdullah KG, Hatanpaa KJ, Walker JM (2021). Prognostic value of isolated TERT promoter mutation in grade 2 and 3 IDH-wildtype astrocytomas. J Neuropathol Exp Neurol.

[96] Richardson TE, Sathe AA, Kanchwala M, Jia G, Habib AA, Xiao G, Snuderl M, Xing C, Hatanpaa KJ (2018). Genetic and epigenetic features of rapidly progressing IDH-mutant astrocytomas. J Neuropathol Exp Neurol.

[97] Richardson TE, Sathe AA, Xing C, Mirchia K, Viapiano MS, Snuderl M, Abdullah KG, Hatanpaa KJ, Walker JM (2021). Molecular signatures of chromosomal instability correlate with copy number variation patterns and patient outcome in IDH-mutant and IDH-wildtype astrocytomas. J Neuropathol Exp Neurol.

[98] Richardson TE, Snuderl M, Serrano J, Karajannis MA, Heguy A, Oliver D, Raisanen JM, Maher EA, Pan E, Barnett S (2017). Rapid progression to glioblastoma in a subset of IDH-mutated astrocytomas: a genome-wide analysis. J Neurooncol.

[99] Richardson TE, Williams M, Galbraith K, Mirchia K, Kumar A, Xing C, Walker JM (2020). Total copy number variation, somatic mutation burden, and histologic grade correlate with clinical outcome in oligodendroglioma. Clin Neuropathol.

[100] Rosswog C, Bartenhagen C, Welte A, Kahlert Y, Hemstedt N, Lorenz W, Cartolano M, Ackerman S, Perner S, Vogel W (2021). Chromothripsis followed by circular recombination drives oncogene amplification in human cancer. Nat Genet.

[101] Sahm F, Koelsche C, Meyer J, Pusch S, Lindenberg K, Mueller W, Herold-Mende C, von Deimling A, Hartmann C (2012). CIC and FUBP1 mutations in oligodendrogliomas, oligoastrocytomas and astrocytomas. Acta Neuropathol.

[102] Sato H, Uzawa N, Takahashi K, Myo K, Ohyama Y, Amagasa T (2010). Prognostic utility of chromosomal instability detected by fluorescence in situ hybridization in fine-needle aspirates from oral squamous cell carcinomas. BMC Cancer.

[103] Satomi K, Fujimoto K, Arita H, Yamasaki K, Matsushita Y, Nakamura T, Miyakita Y, Umehara T, Kobayashi K, Tamura K (2021). DNA methylome analysis suggested the presence of “true” IDH-wildtype lower-grade gliomas. Neurooncol Adv.

[104] Schafer N, Gielen GH, Rauschenbach L, Kebir S, Till A, Reinartz R, Simon M, Niehusmann P, Kleinschnitz C, Herrlinger U (2019). Longitudinal heterogeneity in glioblastoma: moving targets in recurrent versus primary tumors. J Transl Med.

[105] Shirahata M, Ono T, Stichel D, Schrimpf D, Reuss DE, Sahm F, Koelsche C, Wefers A, Reinhardt A, Huang K (2018). Novel, improved grading system(s) for IDH-mutant astrocytic gliomas. Acta Neuropathol.

[106] Shoshani O, Brunner SF, Yaeger R, Ly P, Nechemia-Arbely Y, Kim DH, Fang R, Castillon GA, Yu M, Li JSZ (2021). Chromothripsis drives the evolution of gene amplification in cancer. Nature.

[107] Snuderl M, Eichler AF, Ligon KL, Vu QU, Silver M, Betensky RA, Ligon AH, Wen PY, Louis DN, Iafrate AJ (2009). Polysomy for chromosomes 1 and 19 predicts earlier recurrence in anaplastic oligodendrogliomas with concurrent 1p/19q loss. Clin Cancer Res.

[108] Sparks AB, Morin PJ, Vogelstein B, Kinzler KW (1998). Mutational analysis of the APC/beta-catenin/Tcf pathway in colorectal cancer. Cancer Res.

[109] Stirling PC, Bloom MS, Solanki-Patil T, Smith S, Sipahimalani P, Li Z, Kofoed M, Ben-Aroya S, Myung K, Hieter P (2011). The complete spectrum of yeast chromosome instability genes identifies candidate CIN cancer genes and functional roles for ASTRA complex components. PLoS Genet.

[110] Stoyanov GS, Dzhenkov DL (2018). On the concepts and history of glioblastoma multiforme - morphology, genetics and epigenetics. Folia Med.

[111] Suwala AK, Stichel D, Schrimpf D, Kloor M, Wefers AK, Reinhardt A, Maas SLN, Kratz CP, Schweizer L, Hasselblatt M (2021). Primary mismatch repair deficient IDH-mutant astrocytoma (PMMRDIA) is a distinct type with a poor prognosis. Acta Neuropathol.

[112] Takami S, Kawasome C, Kinoshita M, Koyama H, Noguchi S (2001). Chromosomal instability detected by fluorescence in situ hybridization in Japanese breast cancer patients. Clin Chim Acta.

[113] Thompson LL, Jeusset LM, Lepage CC, McManus KJ (2017). Evolving therapeutic strategies to exploit chromosome instability in cancer. Cancers (Basel).

[114] Thompson SL, Bakhoum SF, Compton DA (2010). Mechanisms of chromosomal instability. Curr Biol.

[115] Tirosh I, Venteicher AS, Hebert C, Escalante LE, Patel AP, Yizhak K, Fisher JM, Rodman C, Mount C, Filbin MG (2016). Single-cell RNA-seq supports a developmental hierarchy in human oligodendroglioma. Nature.

[116] Tischkowitz MD, Chisholm J, Gaze M, Michalski A, Rosser EM (2004). Medulloblastoma as a first presentation of fanconi anemia. J Pediatr Hematol Oncol.

[117] Venteicher AS, Tirosh I, Hebert C, Yizhak K, Neftel C, Filbin MG, Hovestadt V, Escalante LE, Shaw ML, Rodman C (2017). Decoupling genetics, lineages, and microenvironment in IDH-mutant gliomas by single-cell RNA-seq. Science.

[118] Vishwakarma R, McManus KJ (2020). Chromosome instability; implications in cancer development, progression, and clinical outcomes. Cancers (Basel).

[119] von Deimling A, Ono T, Shirahata M, Louis DN (2018). Grading of diffuse astrocytic gliomas: a review of studies before and after the advent of IDH testing. Semin Neurol.

[120] von Hansemann D (1890). Über asymmetrische Zellteilung in Epithelkrebsen und deren biologische Bedeutung. Virchows Arch Patholog Anat.

[121] Walther A, Houlston R, Tomlinson I (2008). Association between chromosomal instability and prognosis in colorectal cancer: a meta-analysis. Gut.

[122] Yan H, Parsons DW, Jin G, McLendon R, Rasheed BA, Yuan W, Kos I, Batinic-Haberle I, Jones S, Riggins GJ (2009). IDH1 and IDH2 mutations in gliomas. N Engl J Med.

[123] Ye CJ, Sharpe Z, Alemara S, Mackenzie S, Liu G, Abdallah B, Horne S, Regan S, Heng HH (2019). Micronuclei and genome chaos: changing the system inheritance. Genes (Basel).

[124] Yip S, Butterfield YS, Morozova O, Chittaranjan S, Blough MD, An J, Birol I, Chesnelong C, Chiu R, Chuah E (2012). Concurrent CIC mutations, IDH mutations, and 1p/19q loss distinguish oligodendrogliomas from other cancers. J Pathol.

[125] Yoda RA, Marxen T, Longo L, Ene C, Wirsching HG, Keen CD, Holland EC, Cimino PJ (2019). Mitotic index thresholds do not predict clinical outcome for IDH-mutant astrocytoma. J Neuropathol Exp Neurol.

[126] Yoo JW, Seo KW, Jang SJ, Oh YM, Shim TS, Kim WS, Lee DS, Lee SD, Choi CM (2010). The relationship between the presence of chromosomal instability and prognosis of squamous cell carcinoma of the lung: fluorescence in situ hybridization analysis of paraffin-embedded tissue from 47 Korean patients. J Korean Med Sci.

[127] Zehir A, Benayed R, Shah RH, Syed A, Middha S, Kim HR, Srinivasan P, Gao J, Chakravarty D, Devlin SM (2017). Mutational landscape of metastatic cancer revealed from prospective clinical sequencing of 10,000 patients. Nat Med.

[128] Zhao Y, Carter R, Natarajan S, Varn FS, Compton DA, Gawad C, Cheng C, Godek KM (2019). Single-cell RNA sequencing reveals the impact of chromosomal instability on glioblastoma cancer stem cells. BMC Med Genom.

